# Impact of HOXB7 overexpression on human adipose-derived mesenchymal progenitors

**DOI:** 10.1186/s13287-019-1200-6

**Published:** 2019-03-19

**Authors:** Elisabetta Manuela Foppiani, Olivia Candini, Ilenia Mastrolia, Alba Murgia, Giulia Grisendi, Anna Valeria Samarelli, Giulia Boscaini, Lucrezia Pacchioni, Massimo Pinelli, Giorgio De Santis, Edwin M. Horwitz, Elena Veronesi, Massimo Dominici

**Affiliations:** 1 Division of Oncology, Department of Medical and Surgical Sciences for Children and Adults, University-Hospital of Modena and Reggio Emilia, Via del Pozzo, 71, 41100 Modena, Italy; 2 Division of Plastic Surgery, Department of Medical and Surgical Sciences for Children and Adults, University-Hospital of Modena and Reggio Emilia, Modena, Italy; 3 Rigenerand srl, Modena, Medolla Italy; 4 Aflac Cancer and Blood Disorders Center, Children’s Healthcare of Atlanta and Emory University Department of Pediatrics, Atlanta, GA USA; 5 Technopole of Mirandola TPM, Mirandola, Modena Italy

**Keywords:** MSC, HOXB7, Aging, bFGF

## Abstract

**Background:**

The ex vivo expansion potential of mesenchymal stromal/stem cells (MSC) together with their differentiation and secretion properties makes these cells an attractive tool for transplantation and tissue engineering. Although the use of MSC is currently being tested in a growing number of clinical trials, it is still desirable to identify molecular markers that may help improve their performance both in vitro and after transplantation.

**Methods:**

Recently, HOXB7 was identified as a master player driving the proliferation and differentiation of bone marrow mesenchymal progenitors. In this study, we investigated the effect of HOXB7 overexpression on the ex vivo features of adipose mesenchymal progenitors (AD-MSC).

**Results:**

HOXB7 increased AD-MSC proliferation potential, reduced senescence, and improved chondrogenesis together with a significant increase of basic fibroblast growth factor (bFGF) secretion.

**Conclusion:**

While further investigations and in vivo models shall be applied for better understanding, these data suggest that modulation of HOXB7 may be a strategy for innovative tissue regeneration applications.

## Introduction

Mesenchymal stromal/stem cells (MSC) are progenitor cells that have been isolated and expanded from a wide range of tissues [1–7]. Their ability to differentiate and secrete supporting factors makes them attractive therapeutic tools for regenerative medicine [8]. For these reasons, MSC transplantation has received increased attention in different clinical settings [9, 10]. Progress in preclinical models and recent data obtained from clinical trials have demonstrated the therapeutic potential of MSC in tissue regeneration and repair. However, significant questions remain [11]. In particular, the role of MSC and their biological behavior in different physiological and pathological conditions in humans need to be clarified aiming for a more solid therapeutic impact. For this reason, it is important to dissect the behaviors and functions of MSC in these different contexts to identify the molecular players that may help improve the performance of MSC ex vivo or after transplantation [12].

It is known that aging is a complex process that involves every cell and organ of the body and leads to the degeneration of many functions over a lifespan. Tissues progressively lose their regenerative and homeostatic ability with age. The homeostatic and regenerative activities of these tissues can be attributed to the resident stem cells. The age-related changes may be reflections of a decline in stem cell function [13]. However, the mechanisms underlying the control of the aging process in human cells remain poorly understood, which has an impact on the development of cell-based therapies.

By focusing on epigenetic regulation, we discovered that miR-196 is one of the molecular markers that determine the changes in MSC during aging [14]. Starting from miR-196, we identified HOXB7 as an age-related gene that is inversely correlated with senescence in vitro and in vivo. HOX genes are fundamental components of embryonic patterning and morphogenesis with persistent expression into adulthood. Several studies support the involvement of HOX genes at several levels along the stem cell hierarchy, including positional identity, stem cell self-renewal, and differentiation [15–17]. HOX genes have been investigated in a variety of progenitor cells and it has been suggested their role in proliferation, differentiation, and senescence [18–22].

When we forced HOXB7 expression in primary bone marrow MSC (BM-MSC), we observed that HOXB7 significantly increased cell proliferation by an unclear mechanism, which we originally related to an autocrine bFGF loop. Furthermore, forced HOXB7 expression reduced senescence, suggesting that HOXB7 might impact aging in addition to in vitro senescence. Therefore, in the present study, we investigated whether HOXB7 overexpression affected other well-known MSC sources, such as adipose-derived MSC (AD-MSC) in an attempt to demonstrate the role of HOXB7 in other mesenchymal cell populations. Adipose tissue represents a valid reservoir of mesenchymal progenitors [23–25]. Adipose tissue can be obtained in a significant amount under local anesthesia with minimum patient discomfort and can be easily processed to release large numbers of AD-MSC. Therefore, it represents a suitable tool for tissue regeneration and gene therapy approaches [26]. Thus, the aim of this study has been to analyze the impact of HOXB7 overexpression on AD-MSC investigating whether its influence on AD-MSC is comparable to the one observed with BM-MSC. This would allow a better understanding of whether HOXB7 may impact on a variety of mesenchymal progenitors’ performance.

## Materials and methods

### Isolation and expansion of AD-MSC

AD-MSC were obtained from lipoaspirate specimens (*n* = 3 collected between hips/thighs, abdomen, or gluteal region) from individuals (three Caucasian females ranging from 44 to 66 years old) undergoing liposuction for esthetic purposes at the Plastic Surgery Unit, University-Hospital of Modena and Reggio Emilia. Informed consent was obtained, and the study was approved by the local Ethical Committee. All AD-MSC samples were isolated and expanded, and cumulative population doubling (CPD) was calculated as previously reported [27]. RNA extraction, immunophenotypical analysis, and differentiation assays were performed after four culture passages (P4) as described below.

### MigRI-HOXB7 vector for viral infection

The bi-cistronic murine stem cell virus-derived retroviral vector (pMIGR1) encoding the green fluorescence protein (GFP) was modified to carry HOXB7 (NM_004502.3), as previously described [14]. The coding sequence was amplified from total human RNA using primers with short extensions containing cleavage sites for BglII (5′ end) and EcoRI (3′ end): HOXB7 forward (5′-TATCAGATCTAAATCATCCGGCCAAATTATGAG-3′) and HOXB7 reverse (5′-TATCGAATTCTGCCCTTTCTCCATCCCTCAC-3′). The amplicons were cleaved with BglII and EcoRI and cloned into the BglII and EcoRI sites of the MigR1 (MSCV-GFP) vector. The resulting vector was defined as pMIGR1-HOXB7. The empty MIGR1-GFP vector was used as a control. Retrovirus production was performed using the FLYRD18 packaging cell lines as previously described [26].

### FACS analysis

Cell surface antigen expression was investigated in the control cell population, represented by AD-MSC-GFP and HOXB7-overexpressing AD-MSC. The following panels of monoclonal antibodies were assessed: APC-anti-CD45, PE anti-CD14, PE anti-CD73, and PE anti-HLA-DR (BD Pharmigen, San Diego, CA, USA); APC-anti-CD146 (MACS, Miltenyi Biotec Bergisch Gladbach, Germany); APC-anti-CD90 and APC-anti-CD105 (eBioscience, San Diego, CA, USA); and isotype controls (BD Biosciences and BioLegend, San Diego, CA, USA). Samples were analyzed using the FACSAria III flow cytometer (Becton Dickinson, Franklin Lakes, NJ, USA). The data were analyzed using DIVA software (Becton Dickinson, Franklin Lakes, NJ, USA). Cell cycle distribution was evaluated by propidium iodide (PI) staining. Single suspensions of 1 × 10^6^ AD-MSC-GFP or AD-MSC-HOXB7 at passage 12 were fixed with ice-cold 70% ethanol and stored at − 20 °C overnight. Cells were washed in PBS and resuspended in 200 μl of Cell Cycle Solution containing PI, RNase A, and Triton X-100 (Invitrogen, Molecular Probe, USA). The samples were then incubated for 30 min at room temperature in the dark and analyzed by FACSAria III and DIVA software.

### RNA extraction and cDNA synthesis

Total cellular RNA was isolated using TRIzol® (Invitrogen, Carlsbad, MN, USA) according to the manufacturer’s instructions. First-strand complementary DNA (cDNA) was synthesized from 2 μg total RNA using the RevertAid H minus first-strand cDNA synthesis kit (Fermentas - ThermoFisher, Waltham, MA, USA). The cDNA was quantified using a spectrophotometer (Beckman Coulter DU® 730, Pasadena, CA, USA).

### Quantitative real-time PCR

Quantitative real-time PCR (qRT-PCR) was performed using the Applied Biosystems StepOne™ Real-Time PCR System and the Fast SYBR® Green Master Mix reagent. The qRT-PCR reaction (10 μl) consisted of 50 ng cDNA, Fast SYBR Green Master Mix (Applied Biosystems, Foster City, CA, USA), and 300 nM of the forward and reverse primers. Primer sequences are presented in Table 1. Relative target gene expression levels were calculated by the 2-ΔΔCt (cycle threshold) method using the human β-actin gene as the control gene [28].

**Table 1:** Primers used to perform qRT-PCR

Gene	Primer sequence	Amplified length (bp)
*β-actin*	5′-ACCTTCTACAATGAGCTGCG-3′ (sense) 5′-CCTGGATAGCAACGTACATGG-3′ (antisense)	148
*Ki67*	5′-GTCGTGTCTCAAGATCTAGCTTC-3′ (sense) 5′-GTCATCTGCGGTACTGTCTTC-3′ (antisense)	146
*HOXB7*	5′-CCTGGATGCGAAGCTCAG-3′ (sense) 5′-CGTCAGGTAGCGATTGTAGTG-3′ (antisense)	107
*bFGF*	5′-ACCCTCACATCAAGCTACAAC-3′ (sense) 5′-AAAAGAAACACTCATCCGTAA-3′ (antisense)	141
*ALP*	5′-GATGTGGAGTATGAGAGTGACG-3′ (sense) 5′-GGTCAAGGGTCA GGAGTTC-3′ (antisense)	142
*COL1A1*	5′-CCCCTGGAAAGAATGGAGATG-3′ (sense) 5′-TCCAAACCACTGAAACCTCTG-3′ (antisense)	148
*DCN*	5′-AAAATGCCCAAAACTCTTCAGG-3′ (sense) 5′-GCCCCATTTTCAATTCCTGAG-3′ (antisense)	146
*PPAR-*γ	5′-GAGCCCAAGTTTGAGTTTGC-3′ (sense) 5′-GCAGGTTGTCTTGAATGTCTTC-3′ (antisense)	148
*LPL*	5′-GAAGACTCGTTCTCAGATGCC-3′ (sense) 5′-GAATGGGATGTTCTCACTCTCG-3′ (antisense)	145
*SOX9*	5′-ACTTGCACAACGCCGAG-3′ (sense) 5′-CTGGTACTTGTAATCCGGGTG-3′ (antisense)	140
*COL2A1*	5′-CCTCAAGGATTTCAAGGCAATC-3′ (sense) 5′-ACCCCTTTCACCAGCTTTTC-3′ (antisense)	144
*p21*	5′-GAGGCCGGGATGAGTTGGGAGGAG-3′ (sense) 5′-CAGCCGGCGTTTGGAGTGGTAGAA-3′ (antisense)	221
*p53*	5′-GAGCTGAATGAGGCCTTGGA-3′ (sense) 5′-CTGAGTCAGGCCCTTCTGTCTT-3′ (antisense)	151

### β-Galactosidase staining

pH-dependent senescence-associated β-galactosidase activity (SA-β-gal) was analyzed using the SA-β-gal staining kit (Cell Signaling Technology, Boston, MA, USA). AD-MSC were seeded at 5500/cm^2^ in triplicate in a 6-well plate. When the cells reached 60 to 70% confluence, they were stained for SA-β-gal according to the manufacturer’s instructions. Positive cells were counted in independent fields (*n* = 10) for each sample at × 2.5 magnification.

### ELISA

The bFGF levels in the AD-MSC samples were measured using the Quantikine human bFGF kit (R&D Systems, 614 McKinley Place NE, Minneapolis, MN, USA) according to the manufacturer’s instructions. This assay employs the quantitative sandwich enzyme immunoassay technique.

### Differentiation assays

The differentiation potential of AD-MSC for three lineages was assessed as previously reported [27]. For osteogenic induction, AD-MSC were seeded in conditioning medium for 2 weeks. The osteogenic medium for the first week consisted of α-MEM supplemented with 8% (*v*/*v*) plate lysate (PL) and inducing agents (10 mM β-glycerophosphate, 0.1 mM ascorbic acid-2-phosphate, and 10 nM dexamethasone (Sigma-Aldrich). For the second week, 100 ng/ml rhBMP-2 (Peprotech, UK) was added to the osteogenic medium. Osteogenic differentiation was assessed by von Kossa staining of the cultured cells that were fixed in ice-cold methanol for 2 min, rinsed in distilled water, and incubated with 1% silver nitrate for 30 min under a UV lamp. The stained samples were washed and then visualized at ELISA × 10 magnification using an inverted microscope (Zeiss). The percentage of von Kossa-stained cells was calculated using ImageJ software (http://rsb.info.nih.gov/ij). For chondrogenic differentiation experiments, the cells were pelleted by centrifugation and incubated at 37 °C with 5% CO_2_ in a humidified incubator (lid not closed). To test the chondrogenic differentiation potential of transduced and non-transduced AD-MSC, the cells were maintained in DMEM high glucose (Gibco by Life Technologies, Paisley, UK) containing 1% penicillin/streptomycin (Carlo Erba, Rodano (MI), Italia); 500 ng/mL BMP-6 and 10 ng/mL TGF-β (Peprotech, Rocky Hill, NJ, USA); BD ITS™ + Premix (BD Biosciences); 100 nM dexamethasone, 0.2 mM ascorbic acid-2-phosphate, and 40 μg/mL proline (Sigma-Aldrich, St. Louis, MO, USA); and 100 μg/mL sodium pyruvate (Biochrom). After 21 days of induction, the samples were inserted into paraffin blocks and cut into 4-μm sections for Safranin O and Fast Green staining. Slides were evaluated using light microscopy (Axiovert 200 M, Zeiss).

To test the adipogenic differentiation potential of the transduced and non-transduced AD-MSC, the cells were maintained in DMEM low glucose (Gibco by Life Technologies, Paisley, UK) containing 1% glutamine (Lonza, Verviers, Belgium), 1% penicillin/streptomycin (Carlo Erba, Rodano (MI), Italia), 10% rabbit serum (EuroClone, Pero (MI), Italy), 5% horse serum (EuroClone, Pero (MI), Italy) and 1 μM dexamethasone, 60 μM indomethacin, 10 μM recombinant human insulin, and 0.5 mM isobutyl methyl xanthine (Sigma, St. Louis, MO, USA). All culture plates were incubated at 37 °C with 5% CO_2_. The cells were induced for 10 days, and lipid droplets were detected by Oil Red O staining (Sigma, St. Louis, MO, USA) using light microscopy (Axiovert 200 M, Zeiss).

### Immunohistochemistry

Chondrogenic differentiation was tested by immunohistochemistry by anti-aggrecan (ACAN) and anti-Collagen 2A1 (Col2A1) antibodies. Paraffin sections (4-mm thick) were dehydrated and stained by rabbit anti-human Col2A1 (1:75; Novus Biologicals, Centennial, CO, USA) and by rabbit anti-human ACAN (1:1000; Abcam, Cambridge, UK) combined with a goat anti-rabbit biotinylated secondary antibody (Ab) (1:200; Vector Laboratories, Burlingame, CA, USA) and detected by an avidin-biotin-horseradish peroxidase system (Vector Laboratories). Antigen retrieval was performed by proteinase K, 1× (Promega Corporation, Madison, WI, USA) treatment for 5 min at room temperature, blocking nonspecific binding with 10% new-calf serum blocking reagent (Sigma-Aldrich, St. Louis, MO, USA). Primary antibodies in 0.1% bovine serum albumin (Sigma-Aldrich) and 10% normal goat serum (Vector Laboratories) were applied overnight. After secondary Ab (Vector Laboratories) incubation and quenching, slides were incubated with Vectastain ABC (Vector Laboratories) as suggested by manufacturer instructions. Color development was performed by Peroxidase Substrate Kit DAB (Vector Laboratories) and slides were counterstained with Mayer’s Hematoxylin (Sigma-Aldrich). Isotype control was stained with only secondary Ab. Stained slides were then examined by Zeiss Axioskop (Zeiss) and photomicrographs were acquired by Axiocam IcC3 color camera and Axiovision 4.82 software visualization (Zeiss).

### Statistical analysis

Data are expressed as the mean value ± standard error of the mean (SEM) of three independent samples. Statistical significance was determined using the two-tailed Student *t* test and ANOVA (GraphPad Prism 7, San Diego, CA, USA). A *p* value of < 0.05 indicates statistical significance.

## Results

### HOXB7 overexpression affects main morphological features sparing the phenotype of AD-MSC

**Fig. 1:**
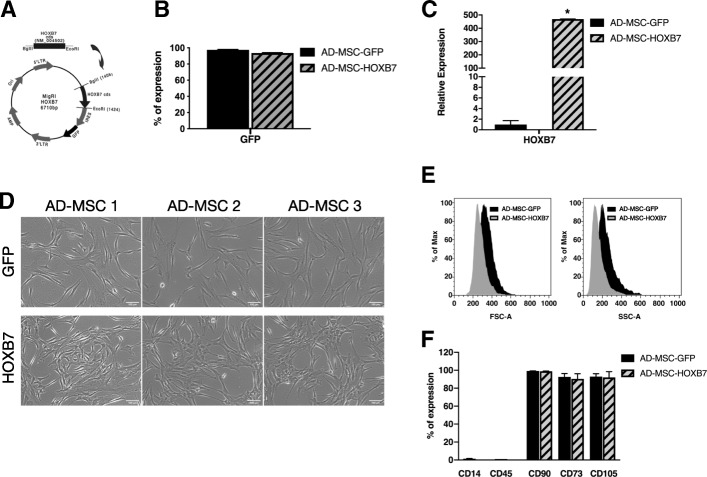
HOXB7 overexpression leads to morphological changes of AD-MSC without affecting the immunophenotypic profile. **a** MigR1-HOXB7 vector. **b** Expression of green fluorescent protein (GFP) detected by FACS in three independent AD-MSC samples (AD-MSC 1, AD-MSC 2, AD-MSC 3) transduced with either empty vector (MSC-GFP) or MigR1-HOXB7 (MSC-HOXB7). **c** Relative HOXB7 expression in AD-MSC-HOXB7 compared to AD-MSC-GFP. Data are the mean of three biological samples, *p value = 0.001. **d** Microscopic morphology of the three different biological samples (AD-MSC 1, AD-MSC 2, AD-MSC 3) either overexpressing HOXB7 (lower panel) or transduced with empty vector (GFP, upper panel), scale bar 100 μm. **e** Representative sample analyzed for physical parameters by FACS. **f** Expression of surface markers CD14, CD45, CD90, CD73, and CD105 by FACS analysis in AD-MSC-GFP and AD-MSC-HOXB7

To investigate whether overexpression of HOXB7 could influence the performance of AD-MSC, three independent AD-MSC samples (AD-MSC 1, AD-MSC 2, and AD-MSC 3) were transduced with either a vector encoding full-length human HOXB7 (MIGR1-HOXB7) (Fig. 1a) or an empty control vector (MIGR1-GFP). In all cases, high transduction efficiencies were observed as indicated by a robust GFP signal (> 98% GFP positivity by FACS; Fig. 1b).

HOXB7 expression was quantified in all the transduced AD-MSC by qRT-PCR. HOXB7 expression levels were 470-fold higher in AD-MSC-HOXB7 compared to control AD-MSC-GFP (Fig. 1c). After transduction, the cells were expanded, and their morphology and antigenic profiles evaluated. HOXB7-related morphological changes were observed in all three samples. In particular, AD-MSC-HOXB7 grew to a higher density and retained a smaller size compared to AD-MSC-GFP, which had larger cytoplasmic bodies (Fig. 1d). To validate these observations, the main AD-MSC physical parameters, such as forward scatter (FSC) and side scatter (SSC), were analyzed by FACS. AD-MSC-HOXB7 had a reduced size (FSC) with lower internal complexity (SSC) (Fig. 1e), suggesting a change in the AD-MSC cell structure, which was previously observed for BM-MSC. However, the AD-MSC immunophenotype was not influenced by HOXB7 overexpression. The cells were positive for the main mesenchymal markers (e.g., CD90, CD105, and CD73) and lacked the expression of hematopoietic antigens, including CD45 and CD14 (Fig. 1f). Collectively, these data indicated that in vitro amplified AD-MSC that overexpressing HOXB7 were morphologically changed in vitro but maintained the typical surface AD-MSC fingerprint.

### HOXB7 reduces senescence and facilitates the proliferation of AD-MSC ex vivo

**Fig. 2:**
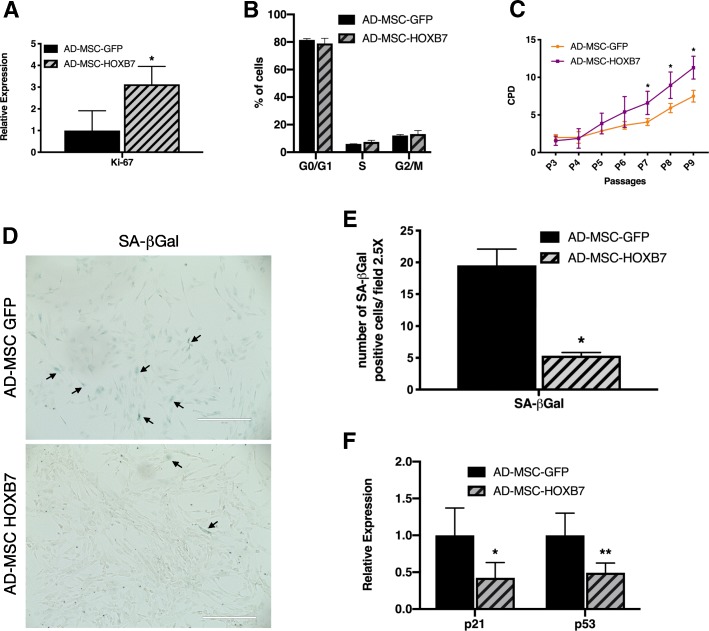
HOXB7 reduces senescence and increases the proliferation potential of AD-MSC ex vivo. **a** Relative Ki67 expression in AD-MSC-HOXB7 compared to AD-MSC-GFP at passage 9, *p value = 0.02. **b** Histogram indicating the percentages of the cells in G0/G1, S, and G2/M phases by FACS analyses. **c** Cumulative population doubling of AD-MSC-HOXB7 and AD-MSC-GFP calculated from passages 3 to 9. **d** Representative SA-β-Gal staining of AD-MSC-GFP (upper panel) and AD-MSC-HOXB7 (lower panel) performed at passage 13 at 4× high power field (*n* = 10) (EVOS™ FL Auto Imaging System). Scale bar 400 μm. (**e**) Quantification of SA-β-Gal positive cells at 2.5× high power field (*n* = 10), *p value = 1.92E−09. **f** Relative expression of senescence-related genes p21 and p53 by qRT-PCR in AD-MSC-HOXB7 compared to AD-MSC-GFP. Data are the mean of three biological samples, *p value = 0.02, **p value = 0.001

In an attempt to verify the hypothesis that the improved cell performance was due to transduced HOXB7 overexpression, the in vitro cell expansion was monitored by the expression of Ki-67, a known proliferation marker [29, 30]. There was a 3.1-fold increase in Ki-67 expression in AD-MSC-HOXB7 compared to AD-MSC-GFP as determined by qRT-PCR, which suggests that HOXB7 might play a role in the regulation of AD-MSC proliferation (Fig. 2a). To investigate whether the increased proliferative potential of MSC HOXB7 cells could be due to change in the cell cycle phases, a cell cycle analysis was implemented (Fig. 2b). The data revealed an increase, although non-significant, of the S/G2/M phases in AD-MSC-HOXB7 versus AD-MSC-GFP. Long-term in vitro proliferation studies instead showed that AD-MSC-HOXB7 had a greater proliferation capacity starting from passage 7 (four passages after viral infection), which indicated a significantly higher performance compared to the AD-MSC-GFP (Fig. 2b). The effect of HOXB7 overexpression on the senescence of AD-MSC was evaluated using SA-ß-Gal staining. The number of SA-β-Gal positive cells was higher for the AD-MSC-GFP samples compared to the AD-MSC-HOXB7 samples (Fig. 2d, e). In order to validate these findings accounting for known markers of MSC senescence [31], p21 and p53 were additionally assessed by qRT-PCR. Interestingly, AD-MSC-HOXB7 significantly downregulated p21 and p53 (Fig. 2f), confirming a relation between HOXB7 expression and a reduced senescence. These findings collectively suggest that HOXB7 could alter AD-MSC performance by simultaneously increasing proliferation and reducing senescence involving known molecular pathways.

### HOXB7 overexpression increases bFGF secretion by AD-MSC

**Fig. 3:**
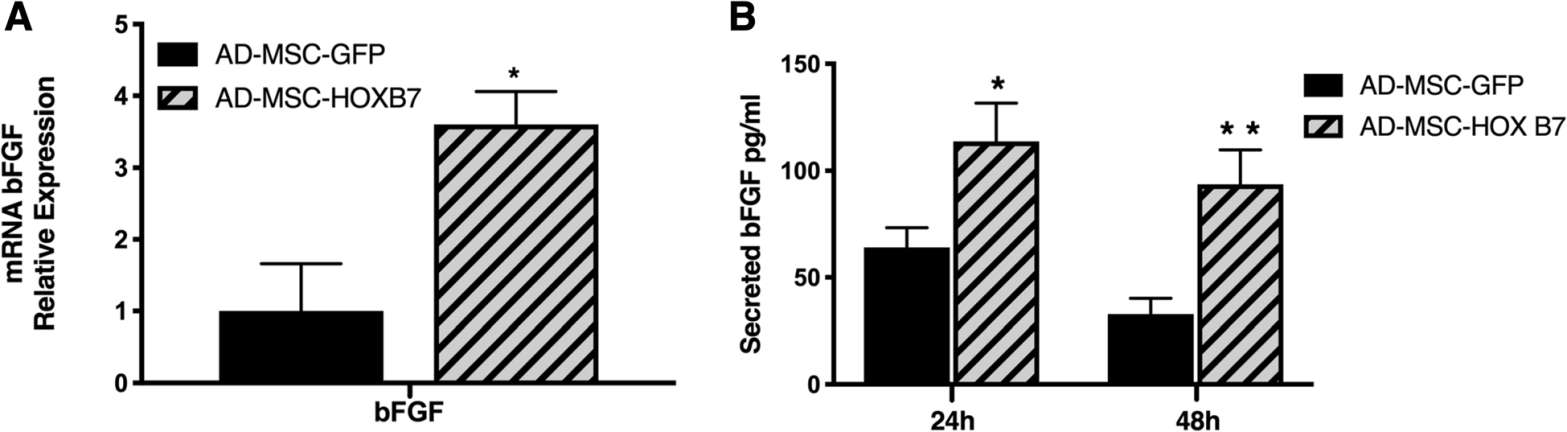
HOXB7 increases levels of bFGF secretion by AD-MSC. **a** Relative bFGF expression in AD-MSC-HOXB7 compared to AD-MSC-GFP. Data are the mean of three biological samples, *p value = 0.003. **b** Secreted bFGF in the supernatants of AD-MSC-GFP and AD-MSC-HOXB7 after 24 or 48 h in culture by ELISA experiment. Data are the mean of three biological samples, *p value = 0.03 HOXB7 versus GFP at 24 h and **p value = 0.01 versus GFP at 48 h

To understand the proliferative properties of AD-MSC-HOXB7, we focused on bFGF as the pivotal MSC mitogen and known transcriptional target of HOXB7 in cancer [32] bFGF was highly expressed in AD-MSC-HOXB7 (Fig. 3a), which was confirmed by measuring the levels of secreted bFGF protein in the AD-MSC-HOXB7 supernatants after 24 or 48 h in culture (Fig. 3b). These data indicated a possible role for bFGF in the changes observed with the AD-MSC populations, which is consistent with the data for BM-MSC [14].

### HOXB7 influences AD-MSC adipogenic differentiation at the molecular level

**Fig. 4:**
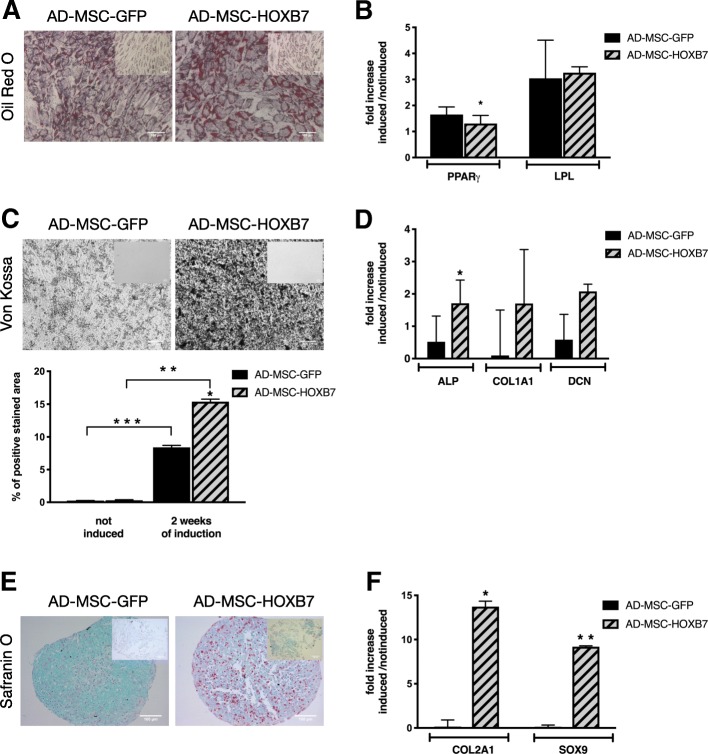
HOXB7 does not alter AD-MSC adipogenic differentiation but enhance osteogenic potential and favors the chondrogenic potential of AD-MSC. **a** Adipogenic differentiation after 10 days of induction visualized by Oil Red O staining in AD-MSC-HOXB7 and AD-MSC-GFP; uninduced controls (inset). Scale bar 100 μm. (**b**) Histograms show the mRNA expression of the adipogenic markers PPAR-γ and LPL after induction in AD-MSC-GFP and AD-MSC-HOXB7. Data are the mean of three biological samples, *p value = 4.90E-06. **c** Von Kossa staining after osteogenic induction of AD-MSC-GFP and AD-MSC-HOXB7 (uninduced control, inset). Scale bar 100 μm. ImageJ quantification of the positive stained area on the right; *p value = 2.3E−32 HOXB7 versus GFP after 10 days of induction, **p value = 1.9E−61, and ***p value = 2.4E−80. **d** mRNA expression of osteogenic markers ALP (*p value = 0.02), COL1A1, and DCN. Histograms show the relative expression of the markers in AD-MSC-GFP and AD-MSC-HOXB7 after induction. Data are the mean of three biological samples. **e** Chondrogenic differentiation after 21 days of induction visualized by Safranin O/Fast Green staining in AD-MSC-GFP and AD-MSC-HOXB7; uninduced controls (inset). Scale bar 100 μm. **f** Histograms show the mRNA expression of adipogenic markers COL2A1 and SOX9 in AD-MSC-GFP and AD-MSC-HOXB7 after induction. Data are the mean of three biological samples, *p value = 0.01 and 0.00008, respectively

Having defined the AD-MSC phenotype, we next assessed the human AD-MSC adipogenic differentiation potential. AD-MSC-HOXB7 were incubated with adipogenic induction media. After 10 days, both AD-MSC-GFP and AD-MSC-HOXB7 differentiated towards the adipogenic lineage as indicated by the round lipid droplets inside the cells and positive Oil Red O staining (Fig. 4a). As expected, no staining was observed in the undifferentiated controls (Fig. 4a). Since there was no difference in the adipogenic differentiation of AD-MSC-HOXB7 and AD-MSC-GFP, we performed qRT-PCR to evaluate the expression of adipocyte-specific genes (Fig. 4b). Interestingly, there were significantly lower levels of PPAR-γ in the AD-MSC-HOXB7, indicating a less committed phenotype after induction (Fig. 4b).

### HOXB7 enhances AD-MSC osteogenic potential

Since AD-MSC can differentiate into bone matrix-producing cells and HOX genes are associated with prenatal skeletal development [33], we evaluated whether HOXB7 overexpression could alter the mineralizing potential of AD-MSC using an rhBMP-2-based in vitro assay. After 2 weeks of induction, von Kossa staining was performed on induced and uninduced AD-MSC. The induced AD-MSC-HOXB7 had a greater mineralization potential compared to the induced AD-MSC-GFP with more than a two-fold increase in osteogenic commitment (Fig. 4c). These cells also had a significant increase in the expression of a key osteogenic marker, alkaline phosphatase (ALP), by qRT-PCR. Expression of two additional osteogenic genes (Collagen 1A1 and Decorin) was also increased, but the changes did not reach statistical significance, likely due to the large variability among the biological replicates (Fig. 4d). Overall, these data indicated increased in vitro mineralization and ALP following overexpression of HOXB7.

### HOXB7 dramatically increases AD-MSC chondrogenesis

**Fig. 5:**
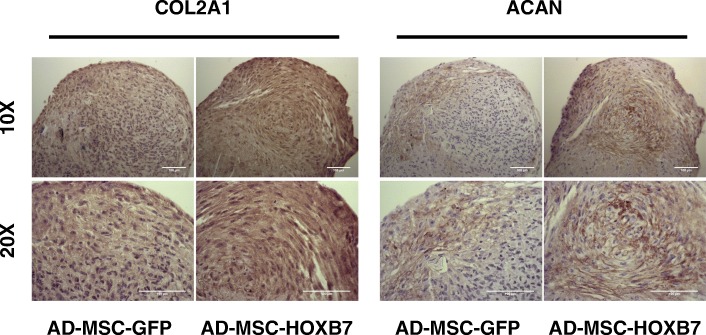
Representative images of AD-MSC-HOXB7 and AD-MSC-GFP pellet cultures stained by the anti-COL2A1 antibody (left panel) and by the anti-ACAN antibody (right panel) after 21 day of chondrogenic induction. Scale bar 100 μm

The pellet culture system was used to evaluate whether HOXB7 overexpression might alter the chondrogenic differentiation of AD-MSC. AD-MSC-HOXB7 and AD-MSC-GFP were induced for 21 days and then stained with Safranin O to detect acid proteoglycan, which is present in cartilage tissues as indicated by a typical orange-red color [34]. Histological sections showed a greater accumulation of cartilage-like extracellular matrix and proteoglycan in the induced AD-MSC-HOXB7 compared to the AD-MSC-GFP. As expected, no staining was observed in undifferentiated cells (Fig. 4e). To confirm the histological data, expression levels of the typical chondrogenic markers, SOX9 and Collagen 2A1 (COL2A1), were measured by qRT-PCR. The AD-MSC-HOXB7 had higher expression of both genes compared to the AD-MSC-GFP (Fig. 4f). To further validate the greater chondrogenic commitment of AD-MSC-HOXB7, we additionally analyzed COL2A1 and ACAN, as mature chondrogenic markers (Fig. 5). AD-MSC-HOXB7 pellets expressed more COL2A1 and ACAN than AD-MSC-GFP. All these data support that HOXB7 promotes a robust chondrogenic differentiation in adipose mesenchymal progenitors.

## Discussion

The promising therapeutic properties of AD-MSC have been evaluated in several fields of regenerative medicine [8, 11]. Over the last several years, progress has been made in the isolation and morphological characterization of AD-MSC and in defining their molecular features and differentiation potential [35, 36]. While deeper preclinical and clinical studies are still required, it has been demonstrated how AD-MSC can mediate their therapeutic effects although their regenerative potential can vary [36, 37]. In particular, human and animal aging may negatively impact AD-MSC properties [38, 40]. The molecular mechanisms behind these events remain largely obscure, precluding the development of counteracting strategies to limit or abrogate aging that may influence proliferation, differentiation, and secretion. Our group has focused on dissecting age- and senescence-related fingerprints to identify key factors that influence stem cell senescence to optimize regenerative medicine and gain a better understanding of human aging. We recently identified changes in HOXB7 levels as an age-related factor and master player that drives BM-MSC proliferation and differentiation [14]. We showed that forced HOXB7 expression in BM-MSC is associated with increased proliferation, reduced senescence, and increased osteogenesis that counteracts the aging that occurs in human and animal BM-MSC [14, 39, 41–43]. The aim of this current study was to investigate the impact of HOXB7 overexpression on human AD-MSC. AD-MSC samples were isolated from lipoaspirates and then expanded, characterized, and transduced with retroviral vectors encoding HOXB7 or GFP control. The transduction efficiency was more than 98% for both HOXB7 and GFP samples. Interestingly, shortly after transduction, the cells carrying the HOXB7 encoding vector had a reduced proliferation capacity compared to the GFP-only control cells (not shown). This phase of growth was reversible and lasted for 10 days after transduction. After this period, the HOXB7 overexpressing AD-MSC cells were more proliferative than the control AD-MSC and possessed morphological changes (e.g., reduction in cell size and internal complexity) that are hallmarks of younger mesenchymal populations [33, 41, 42]. It is well known that human MSC are heterogeneous with differently shaped and sized cells; the large ones propagated very slowly and the small ones more rapidly [44]. Thus, the cell size has been considered a relevant parameter in MSC biology: the large cells are referred to as mature MSC and the smaller ones are referred to as recycling stem (RS) cells. In addition, MSC aging has been associated with an increase in cell size [45]. While further investigations shall be demanded to better clarify the impact of HOXB7 in MSC size, we may speculate that the higher cellular performance identified in the AD-MSC-HOXB7 compared to AD-MSC-GFP may be also related with this change in morphology. The molecular analyses also demonstrated a substantial increase in Ki-67 levels after HOXB7 overexpression. While this was not completely aligned with a non-significant increase S/G2/M phases of AD-MSC-HOXB7, it could be that HOXB7 may play a different role in primary MSC compared to the tumor cell lines where it acts as oncogene dramatically increasing cell proliferation [46, 47]. To our knowledge, there have been no other reports evaluating the overexpression of HOX genes or HOXB family genes to modify adipose progenitors ex vivo. Therefore, with the limitation of an in vitro approach, our cells represent a novel tool for potential therapeutic applications based on adult progenitors. It is well known that HOX genes play an essential role in prenatal development [33]. However, little information is available on the role of HOX genes in post-natal life (e.g., adult stem cell regulation). In this respect, HOXB genes have been studied in other human progenitor compartments, providing initial evidence of their regulatory functions that might allow the development of therapeutic strategies to optimize stem cell self-renewal and commitment [22, 48]. For example, HOXB4 overexpression can enhance the ex vivo expansion of hematopoietic progenitors and promote their in vivo regenerative potential by multiple transcriptional activities, leading to the modulation of several target genes [49]. Similarly, when we overexpressed HOXB7 in BM-MSC, we observed a decrease in cell size, increased Ki-67 expression, and decreased senescence with no changes in their immunophenotypical profile or indications of malignant transformation [14].

The role of the HOX genes in post-natal life was originally confirmed by Wagner et al., who found that the expression of many HOX genes was regulated during aging with most of these genes age-repressed (e.g., HOXA5, HOXB3, and HOXB7) or age-induced (e.g., HOXA7, HOXB5, and HOXB6) in MSC and hematopoietic progenitor cells, respectively [22]. Thus, HOX genes may not only function in prenatal morphogenesis and differentiation but also during post-natal aging. This has been here confirmed by the significant downregulation of p21 and p53, as reported by modified AD-MSC expressing hTERT [50].

Based on our previous studies with BM-MSC and other previously reported data on the role of HOXB7 in regulating bFGF expression [14, 15, 32], we focused on bFGF mRNA and protein expression in the current study. HOXB7 overexpressing AD-MSC secreted significantly higher levels of bFGF, which is consistent with previously published data on bFGF and suggests that bFGF is a key player that counteracts aging in mesenchymal progenitors [14]. In addition, bFGF appears to be a driver of MSC differentiation, which influences this process towards all three mesenchymal lineages [51–55].

Our data revealed that a higher level of secreted bFGF from AD-MSC-HOXB7 was linked to increased osteogenesis and chondrogenesis but had a negligible effect on adipogenesis. However, there were reduced PPAR-γ levels of AD-MSC-HOXB7 under adipogenic conditions, which might indicate a degree of resistance to adipogenic differentiation. These cells were able to generate lipid droplets in vitro, suggesting the persistence of multipotency in the HOXB7 overexpressing cells.

As demonstrated for BM [14] AD-MSC-HOXB7 showed a greater mineralization potential versus the induced AD-MSC-GFP with more than a two-fold increase in osteogenic commitment and a significant increase of ALP, a key early marker of bone differentiation. The possible role of HOXB7 in MSC osteogenic commitment has also been described by Gao et al., who reported that HOXB7 positively affects the osteogenic potential of BM-MSC through the upregulation of runt-related transcription factor 2 (RUNX2) and activation of bone sialoprotein (BSP) [55, 56]. These data suggest that activation of HOXB7 signaling might favor bone regeneration by those MSC populations that may not be primed to easily generate skeletal tissues [56–58].

Interestingly, in our model, higher chondrogenesis was enhanced by HOXB7 as determined by gene expression and histologic analyses of cartilage markers indicating a higher expression of key chondrogenic markers (e.g., SOX9, COL2A1, and ACAN). This effect is of particular interest for possible therapeutic applications and tissue development (e.g., cartilage and bone). As reported, a chondrogenic pre-induction of MSC may enhance bone quality by endochondral [58, 59]. In a recent study, Janicki et al. showed that bone quality could be enhanced by chondrogenic pre-induction of BM-MSC/beta-TCP constructs in vitro, which triggers differentiation via the endochondral ossification pathway, attracts hematopoietic marrow more efficiently, and allows full ossicle formation [54]. In another experiment, BM-MSC and AD-MSC from 14 different donors were compared. Brocher et al. reported that BM-MSC seeded on beta-TCP scaffolds induced new bone deposition onto the scaffold whereas no bone deposition was observed with AD-MSC 8 weeks after subcutaneous implantation. The AD-MSC results were moderately improved by prolonging the observation period to 12 weeks [58, 59]. Since several studies favored the use of BM-MSC for osteogenesis and chondrogenesis because of their presumable pre-commitment towards these lineages, our results showed that AD-MSC-HOXB7 could overcome this initial disadvantage. Indeed, HOXB7 overexpression seemed to favor chondrogenesis as a precondition for robust osteogenesis. Studies aimed at developing novel tissue engineering strategies that manipulate the secretion of the inducing ligand or developmental signaling pathways have been reported to improve the clinical efficiency of MSC [60, 61]. However, to our knowledge, HOX genes have never been considered in the context of preconditioned cells, which may be relevant for the production of better performing skeletal tissues, such as the cartilage.

## Conclusion

In conclusion, understanding the importance of HOX genes expression and function will be critical for future studies involving MSC for regenerative medicine. Moreover, the identification of possible molecular mechanisms driving aging may contribute to a better understanding of the age-related disease and the generation of tools to increase ex vivo progenitor performance for improved therapeutic benefits.

## Abbreviations

ACAN: Aggrecan
AD: Adipose tissue
ALP: Alkaline phosphatase
bFGF: Basic fibroblast growth factor
BM: Bone marrow
BMP: Bone morphogenetic protein
CD: Cluster of differentiation
COL1A1: Collagen type I alpha 1 chain
COL2A1: Collagen type II alpha 1 chain
CPD: Cumulative population doubling
DCN: DECORIN
FSC: Forward scatter
G-CSF: Granulocyte colony-stimulating factor
GFP: Green fluorescent protein
HOX: Homeobox
hTERT: Human telomerase reverse transcriptase
LPL: Lipoprotein lipase
MSC: Mesenchymal stromal/stem cells
PPAR-γ: Proliferator-activated receptor-gamma
RS: Recycling stem
RUNX2: Runt-related transcription factor 2
SCF: Stem cell factor
SOX9: SRY-box 9
SSC: Side scatter
